# Rhomboid Intercostal Block Combined With Interscalene Nerve Block for Sternoclavicular Joint Reconstruction

**DOI:** 10.31486/toj.20.0020

**Published:** 2021

**Authors:** Chihiro Toda, Rajnish K. Gupta, Hesham Elsharkawy

**Affiliations:** ^1^ Department of Anesthesiology and Pain Management, Cleveland Clinic, Cleveland, OH; ^2^ Department of Anesthesiology, Vanderbilt University Medical Center, Nashville, TN; ^3^ MetroHealth Pain and Healing Center, Associate Professor of Anesthesiology, Case Western University, Outcome Research Consortium, Cleveland Clinic, Cleveland, OH

**Keywords:** *Anesthesiology*, *fascia*, *nerve block*, *pain–postoperative*, *ultrasonography*

## Abstract

**Background:** Rhomboid intercostal block is a newer technique for chest wall analgesia and can be an effective alternative to thoracic epidurals and paravertebral blocks. We performed a rhomboid intercostal block after sternoclavicular joint reconstruction surgery.

**Case Report:** A healthy 26-year-old male who had chronic right sternoclavicular joint instability was scheduled for right medial clavicle resection with sternoclavicular joint allograft reconstruction. We performed a right interscalene single-shot nerve block followed by a rhomboid intercostal block with catheter placement under ultrasound guidance. The patient's pain was well controlled postoperatively with minimal use of opioids.

**Conclusion:** Rhomboid intercostal block with brachial plexus block is a potential option for analgesia after sternoclavicular joint reconstruction surgery.

## INTRODUCTION

Rhomboid intercostal block—an interfascial plane block for chest wall analgesia—was reported in 2016 as a possible alternative to thoracic epidurals and paravertebral blocks.^1,2^ Since then, several cases have demonstrated the efficacy of rhomboid intercostal block for analgesia after thoracic surgeries and mastectomies.^3-6^ A 2020 randomized controlled trial showed that rhomboid intercostal block decreased opioid consumption after mastectomy.^7^ We report the use of rhomboid intercostal block to provide adequate pain relief after sternoclavicular joint reconstruction surgery.

## CASE REPORT

An otherwise healthy 26-year-old male had chronic right sternoclavicular joint instability and was scheduled for right medial clavicle resection with sternoclavicular joint allograft reconstruction combined with a pectoralis major flap. The surgeon planned a longitudinal parasagittal incision over the sternoclavicular joint and pectoralis major muscle just medial to the midclavicular line, which is covered by the C5, T1 to T4 dermatome.

**Figure 1.**
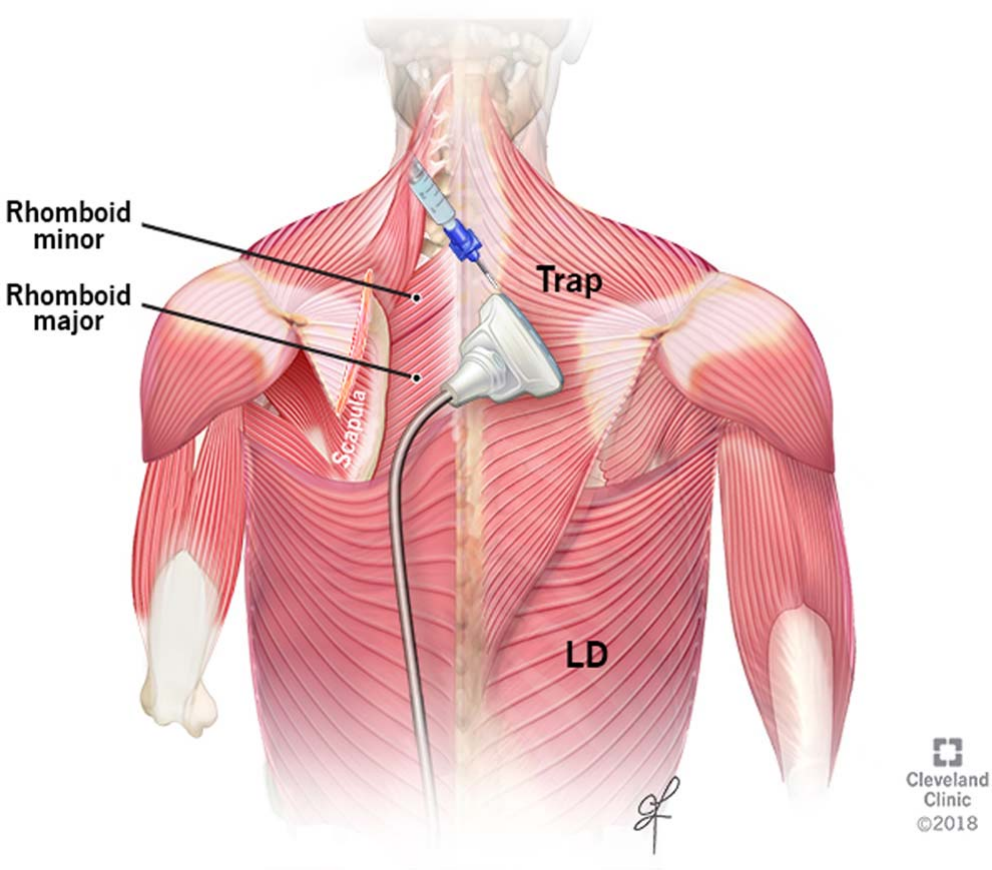
**Illustration shows back anatomy and location of an ultrasound probe for rhomboid intercostal block. Reprinted with permission, Cleveland Clinic Center for Medical Art & Photography © 2021. All Rights Reserved.** LD, latissimus dorsi muscle; Trap, trapezius muscle.

**Figure 2.**
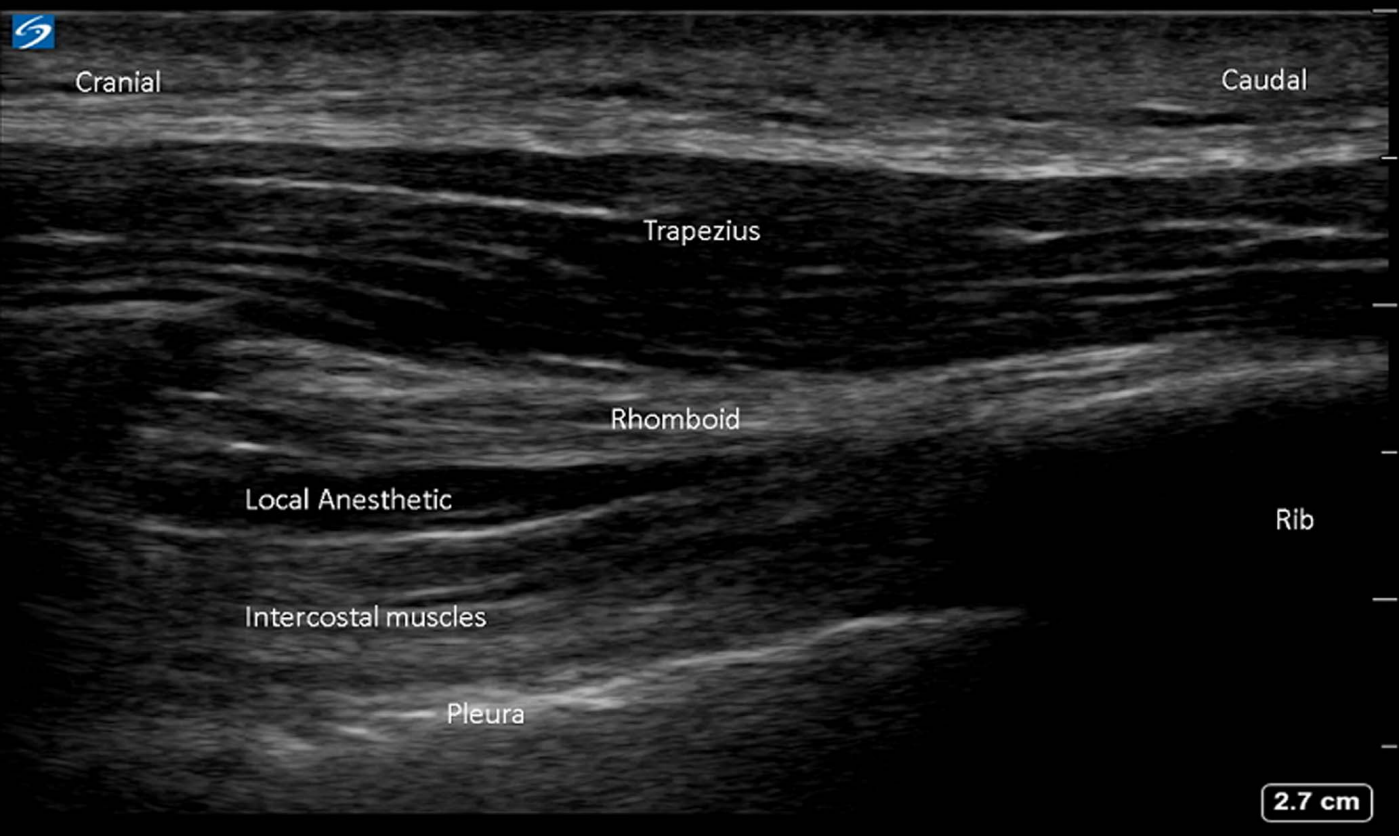
Ultrasound image of rhomboid intercostal block shows the spread of local anesthetic between the rhomboid and intercostal muscles.

Preoperatively, the patient was positioned in the left lateral decubitus position. A right interscalene single-shot nerve block with 10 mL of 0.25% bupivacaine was performed under ultrasound guidance. With the patient in the same position, we performed the rhomboid intercostal block with catheter placement because the majority of the incision dermatomes were T1 to T4. His trapezius muscle, rhomboid muscle, third and fourth ribs, intercostal muscles, and pleura were identified with SonoSite HFL 38xp/13-6 MHz ultrasound (Fujifilm SonoSite, Inc). A 17-g Tuohy needle was advanced in-plane to the ultrasound beam, and 25 mL of 0.25% bupivacaine was injected in the fascial layer between the rhomboid muscle and intercostal muscles (Figure 1). The spread of local anesthetic in a cephalo-caudad direction was confirmed with ultrasound (Figure 2). An Arrow echogenic 19 Ga FlexBlock catheter (Teleflex) was placed after the injection.

The patient received general anesthesia for the surgery and was administered 250 μg of intravenous (IV) fentanyl. Postoperative sensory examination showed decreased sensation from right C7 to T6 in the parasternal region, the anterior midclavicular line, midaxillary line, and posterior scapular line. We started a 0.2% ropivacaine infusion through the rhomboid intercostal block catheter via an ambIT pump (Summit Medical Products, Inc) with the following settings: 8 mL/h of infusion with patient-controlled analgesia of 10 mL of bolus with 60 minutes of lockout time. The patient received 50 μg IV fentanyl 3 times in the postoperative anesthesia care unit (PACU). He reported pain levels at the incision site of 5/10 with movement and 0/10 with rest during his PACU stay.

The patient was discharged home with the pump on the day of surgery. From postoperative day (POD) 1 to POD 4, we called the patient every day to assess whether the pump was working appropriately and whether his pain was controlled. The patient reported that his pain was well controlled, with levels of 0/10 to 4/10, and he required only one dose of 5 mg oxycodone immediate release on POD 3. The patient removed the catheter at home on POD 4 with no difficulty. No major complications were noted.

## DISCUSSION

A rhomboid intercostal block can be a useful tool for managing pain in a challenging surgical location. The sternoclavicular joint is supplied by the medial branch of the supraclavicular nerve that arises from C3 and C4 and travels within the superficial cervical plexus. Traditionally, an interscalene nerve block has been used for clavicle surgery because an interscalene block principally covers the upper and middle trunks of the brachial plexus, originating from C5 to C7, and can provide analgesia for the superficial cervical plexus. However, even if spread from the interscalene nerve block reaches the superficial cervical plexus, the interscalene nerve block is inadequate for surgery involving the medial clavicle and sternoclavicular joint because of the lack of upper thoracic (T1-T4) coverage. A high thoracic epidural would cover the surgical area, but successful placement depends on operator technique, patient body habitus, and spinal pathology.^8^ Thoracic epidural can also cause the significant cardiovascular implications of a high sympathectomy, such as hypotension.^8,9^ A paravertebral block is a reasonable option but can be difficult to place and has variable success rates.^10^ We hypothesized that adding a rhomboid intercostal block to an interscalene nerve block would provide the necessary upper thoracic coverage needed for complete analgesia. The rhomboid intercostal interfascial plane extends medially deep to the erector spinae muscles and laterally deep to the serratus anterior muscle.^2^ In a cadaveric study, injection of dye in this fascial plane stained the lateral cutaneous branches of the intercostal nerves and the dorsal rami of T3 to T9.^2^

Our patient had adequate pain control initially after the surgery and through POD 4 with a single-shot interscalene nerve block combined with a rhomboid intercostal block and catheter infusion. Postoperative physical examination revealed decreased sensation in the parasternal area, which is innervated by anterior branches of intercostal nerves. We did not expect the local anesthetic to spread to the anterior cutaneous branches, but it spread medially to block the main ventral rami and acted as an indirect paravertebral block. This coverage of the ventral rami was seen clinically but not in the cadaver study.^2^

Limitations of this case are the lack of a control case receiving only interscalene nerve block for the same surgery and inadequate information to determine how much the interscalene nerve block contributed to analgesia as the patient was discharged on the same day and followed only by phone interview. More clinical experience and comparison with erector spinae block are needed, but we propose that the rhomboid intercostal block can be an effective addition to the traditional interscalene nerve block for postoperative analgesia in medial clavicle and/or sternoclavicular joint operations.

## CONCLUSION

Rhomboid intercostal block with brachial plexus block is a potential option for analgesia after sternoclavicular joint reconstruction surgery.

## Notes

*This article meets the Accreditation Council for Graduate Medical Education and the American Board of Medical Specialties Maintenance of Certification competencies for Patient Care and Medical Knowledge.*
